# Comparative *Membranome* Expression Analysis in Primary Tumors and Derived Cell Lines

**DOI:** 10.1371/journal.pone.0011742

**Published:** 2010-07-23

**Authors:** Paolo Uva, Armin Lahm, Andrea Sbardellati, Anita Grigoriadis, Andrew Tutt, Emanuele de Rinaldis

**Affiliations:** 1 CRS4 Bioinformatics Laboratory, Parco Scientifico e Tecnologico POLARIS, Pula, Cagliari, Italy; 2 IRBM, Pomezia, Rome, Italy; 3 Breakthrough Breast Cancer Research Unit, King's College London School of Medicine, Guy's Hospital, London, United Kingdom; Deutsches Krebsforschungszentrum, Germany

## Abstract

Despite the wide use of cell lines in cancer research, the extent to which their surface properties correspond to those of primary tumors is poorly characterized. The present study addresses this problem from a transcriptional standpoint, analyzing the expression of membrane protein genes - the *Membranome* – in primary tumors and immortalized *in-vitro* cultured tumor cells. 409 human samples, deriving from ten independent studies, were analyzed. These comprise normal tissues, primary tumors and tumor derived cell lines deriving from eight different tissues: brain, breast, colon, kidney, leukemia, lung, melanoma, and ovary. We demonstrated that the *Membranome* has greater power than the remainder of the transcriptome when used as input for the automatic classification of tumor samples. This feature is maintained in tumor derived cell lines. In most cases primary tumors show maximal similarity in *Membranome* expression with cell lines of same tissue origin. Differences in *Membranome* expression between tumors and cell lines were analyzed also at the pathway level and biological themes were identified that were differentially regulated in the two settings. Moreover, by including normal samples in the analysis, we quantified the degree to which cell lines retain the *Membranome* up- and down- regulations observed in primary tumors with respect to their normal counterparts. We showed that most of the *Membranome* up-regulations observed in primary tumors are lost in the *in-vitro* cultured cells. Conversely, the majority of *Membranome* genes down-regulated upon tumor transformation maintain lower expression levels also in the cell lines. This study points towards a central role of *Membranome* genes in the definition of the tumor phenotype. The comparative analysis of primary tumors and cell lines identifies the limits of cell lines as a model for the study of cancer-related processes mediated by the cell surface. Results presented allow for a more rational use of the cell lines as a model of cancer.

## Introduction

Proteins associated with the cell plasma membranes mediate key processes such as molecular transport, cell adhesion, interaction with the extracellular matrix, signal transduction and cell-to-cell signaling. They have long been recognized to play a crucial role in the genesis and development of cancer, by mediating complex interactions between the tumor cells surface and the surrounding cellular environment [1]. Moreover, this class of proteins is of special relevance in cancer research as it constitutes the target of election of monoclonal antibodies based therapies [2]. In fact a number of monoclonal antibody targeting cell surface proteins have been approved as therapeutics and have consolidated their value in the treatment of cancer [3]. Many studies focusing on cellular processes involving surface properties of cancer cells make use of model cell lines derived from primary tumors. Examples are: i) the identification of tumor specific membrane proteins involved in pathways of adhesion and signaling [4]; ii) the assay of anticancer drugs and antibodies targeting cell surface proteins [5]; iii) the selection of anti-cancer mAbs from antibody libraries using the cell lines as target [6]; iv) cell binding assays and immuno-staining experiments [2]. When using *in*-*vitro* cell models to mimic cancer biology it is important to remember that tumors are complex and heterogeneous systems. They are composed of different cell types, interacting with each other, with the extracellular matrix (ECM) and the surrounding tissue through a complex network of signaling pathways, all mediated by cell surface proteins. In contrast, cell lines consist of homogeneous clonal populations generally lacking interactions with other cell types and instead interacting with an artificial support. Moreover, cell adaptation to *in*-*vitro* microenvironments involves recalibrations of many pathways involving the cell surface, for example by genetic and epigenetic alterations [7], [8], different post-transcriptional regulation [9] and modified signaling networks [10]. Differences in the composition and the functional activity of the cell surface of primary tumors frequently result in different sensitivity to anticancer agents, with cell lines being in general more sensitive to treatments than primary tumors [5]. For these reasons we believe that a quantitative and qualitative assessment of the similarities and differences between the cell surface of primary tumors and related cell lines is of outstanding importance for a more efficacious use of the cell lines as an *in-vitro* cancer model. In fact, despite their wide use, the extent to which the surface properties of cell lines actually correspond to those of the corresponding tumor tissues of origin has been poorly characterized. We addressed this question from a transcriptional standpoint, by performing a meta-analysis of membrane protein gene-expression profiles from ten different studies [8], [11]–[19], all using the same microarray platform. The data set is composed in total of 409 human samples, including normal, primary tumor samples and tumor derived cell lines. Eight different tissue origins are represented: brain, breast, colon, kidney, leukemia, lung, melanoma, and ovary. We defined as the *Membranome* the ensemble of all human genes coding for proteins integral to or covalently associated with the plasma membrane. First, we demonstrated that the *Membranome* expression data have greater power than the rest of the transcriptome when used as input for the automatic classification of tumor samples. This property suggests that most of the gene expression specificity of tumors of different origins resides into the genes codifying for cell surface proteins. This feature is maintained in tumor derived cell lines.

Then we run a systematic comparison between the *Membranome* expression in tumor and cell lines, using three different analytical approaches. The first one is based on the direct comparison of the *Membranome* expression values in primary tumors and cell lines, grouped by tissue of origin. The second focuses on pathways involving the *Membranome* and identifies those pathways differentially regulated in tumors and cognate cell lines. The third analysis quantifies the extent to which cell lines reproduce *Membranome* up- or down-regulation observed in primary tumors with respect to their normal tissue counterparts.

## Results

### Microarray data

Gene expression data on tumor cell lines, primary tumors and normal tissues were integrated from ten independent studies, all based on the Affymetrix HG-U95Av2 array platform (see Methods) (Table 1). The resulting dataset includes 56 cell lines, 294 tumor samples and 59 normal samples representing a total of 8 different tissue origins: brain, breast, colon, kidney, leukemia, lung, melanoma and ovary.

**Table 1:** Microarray datasets on NCI60 cell lines, primary tumors and normal tissues analyzed in this study.

Tissue	Cell Lines[^8^]	Normal tissues	Tumor tissues
Brain	6	9[^17^], [^18^]	21[^15^]
Breast	6	-	19 [^19^]
Colon	7	9[^14^], [^19^]	21 [^19^]
Kidney	8	14[^13^]	11 [^19^]
Leukemia	6	-	72[^11^]
Lung	9	20[^12^]	127[^12^]
Melanoma	8	-	9 [^16^]
Ovary	6	7[^18^], [^19^]	14[^19^]
**Total**	**56**	**59**	**294**

All the gene expression measurements were obtained with Affymetrix HG-U95Av2 arrays.

### Definition of human *Membranome* genes

We defined as the *Membranome* the ensemble of all human genes coding for proteins integral or associated to the plasma membrane. All human genes reported in the NCBI Gene database were surveyed using a combined analysis of the available Gene Ontology annotations [20] and through the Phobius algorithm predicting trans-membrane domains and signal peptides [21].

The resulting human Membranome comprises 4,329 genes (about 17% of human genes) encoding for plasma membrane proteins, neglecting the additional complexity introduced by alternative splicing events or post-translational modifications. Of these genes, 1,701 are represented on the Affymetrix HG-U95A2 microarray platform, common to all data sets considered in the present study (Table S1)

**Figure 1:**
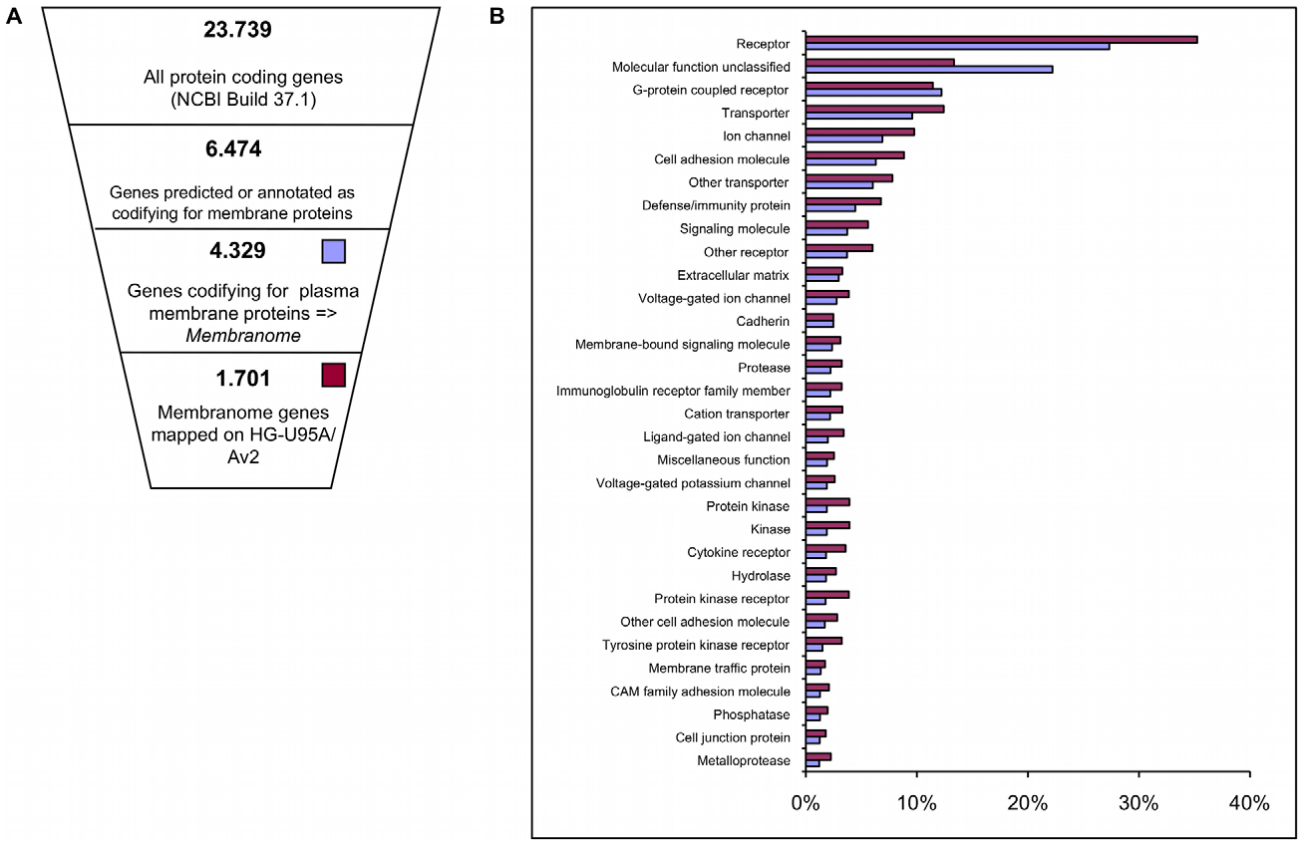
Definition of the Human *Membranome*. A) Schematic representation of the strategy used to identify the Human *Membranome*. Combining annotations (GO), predictions (Phobius) and manual revision we estimate that approximately 17% of human protein coding genes are exposed on the plasma membrane on the cell. 39% of them are represented on the Affymetrix HG-U95A/Av2 array. B) Panther Molecular Function composition of the *Membranome*. The percentage of genes annotated in each category is shown for the complete set of membranome genes (purple) and for the fraction that is represented on the array (blue).

Although the array covers only about 40% of the whole *Membranome* (Fig. 1A), the internal representation of all major functional classes – as defined by Panther [22] - is strictly maintained (Fisher exact test p-value<0.001) (Fig. 1B). Importantly, the class of *Membranome* genes annotated as “molecular unclassified” is under-represented on the array, reflecting a positive bias towards well annotated genes in the process of array design.

### *Membranome* classification power with respect to tissue origin

Much of the biological specificity of different cell and tissue types is conferred by specialized subsets of proteins present on the surface of the cell [23]. A large fraction of these proteins have a structural role, being linked to the cellular cytoskeleton and conferring specific morphologies to different cell types; others mediate the response to external stimulus (e.g. cytokines, growth factors) and/or the interaction with other cells through a variety of molecular mechanisms [24]. To quantify - in terms of gene expression - the contribution of *Membranome* genes in defining the tumor type specificity, we run a parallel classification study on primary tumors and tumor derived cell lines. The classification power of *Membranome* and an equally sized, randomly chosen, set of *Not*-*Membranome* genes, was used as input for the automatic classification of samples with different tumor origin.

**Figure 2:**
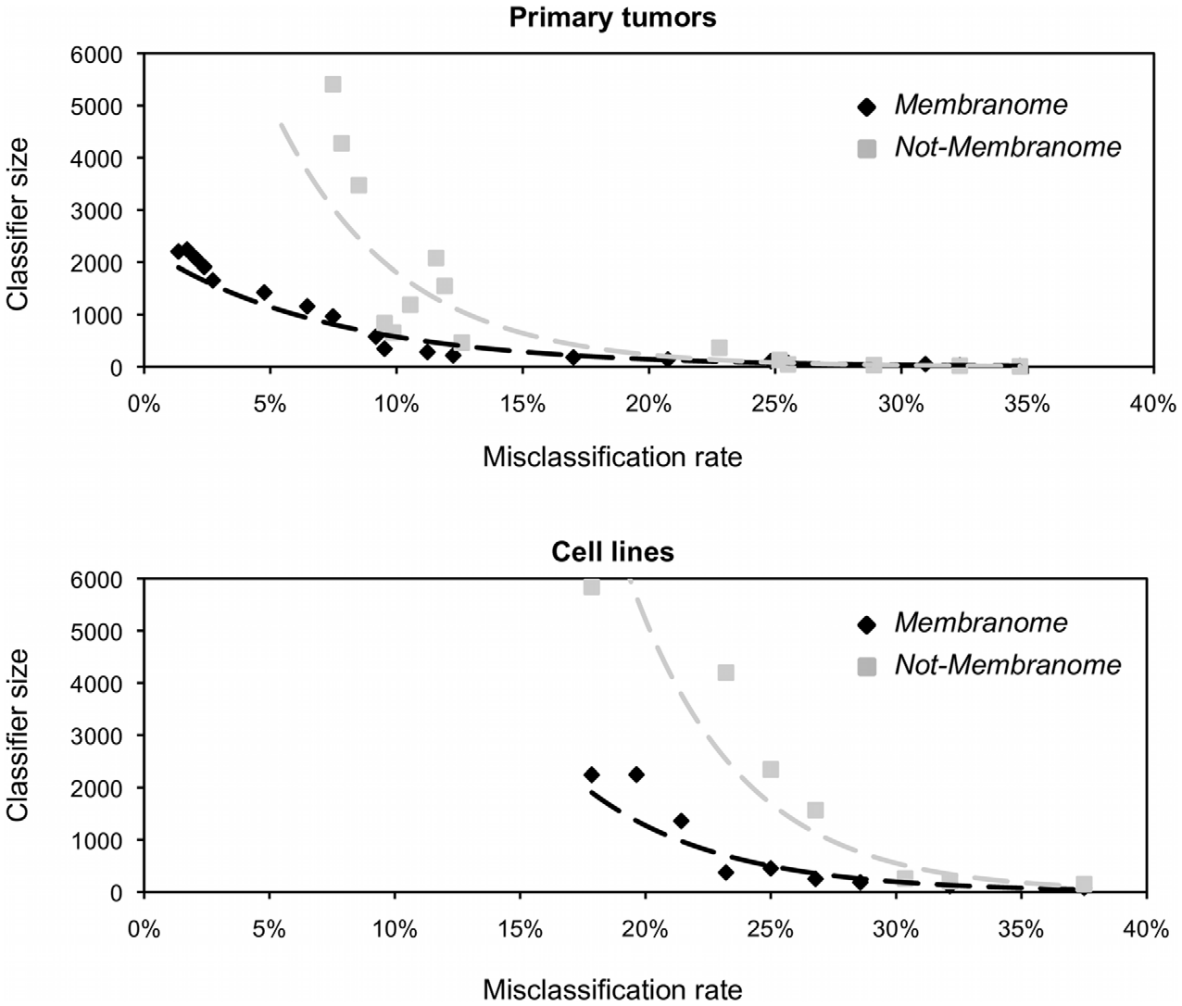
Classification power of *Membranome* genes. Classification power of *Membranome* genes in primary tumors (top panel) and cell lines (bottom panel). PAM algorithm was applied to compute the misclassification rate of both *Membranome* and not- *Membranome* genes using classifiers of increasing size. Dotted lines represent exponential fits of the data points resulting from the analyses. *Membranome* genes showed a lower misclassification rate in classifying both tumor samples and cell lines according to their tissue origin.

The results obtained using classifiers of decreasing size (Fig. 2) show that the *Membranome* genes have a significantly lower misclassification rate - and therefore greater power - in classifying both tumor samples and cell lines according to their tissue of origin. Importantly, the analysis also shows that the misclassification rates obtained for cell lines are significantly higher than those for primary tumors. In both primary tumors and cell lines analyses, the eight tissues of origin analyzed gave rise to comparable frequencies of misclassification. Therefore the obtained misclassification rates cannot be ascribed to specific tissue types.

### Comparison of *Membranome* expression profiles in primary tumors and cell lines

**Figure 3:**
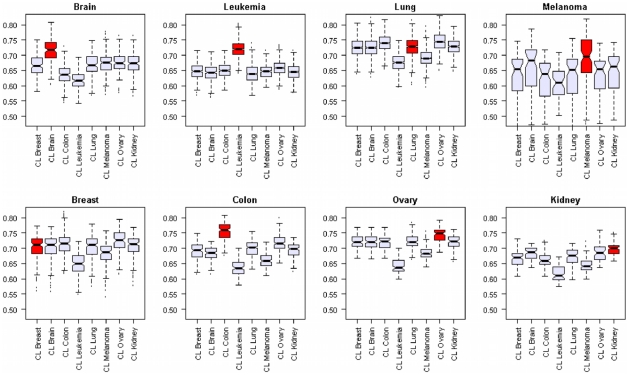
Correlation of Membranome gene expression profiles between primary tumors and cell lines. Each boxplot represents the distribution of correlation coefficients obtained comparing gene expression profiles of cell lines and primary tumors of various tissue origins. The y-axis represents the Pearson's correlation coefficients. The origin of the cell lines is labelled on the x-axis. Boxes corresponding to cell lines and primary tumors with the same tissue origin are labelled in red. Excepted for breast and lung, the primary tumors always showed the highest similarity with their cognate cell lines (t-test p-value<0.01).

To characterize the degree to which cell lines are representative of their tumor of origin with respect to *Membranome* expression, a systematic comparative analysis was performed. *Membranome* gene expression in primary tumors and cell lines were compared using the Pearson's correlation as metrics of similarity, as described in Methods. Correlation values between primary tumors and cell lines, grouped by tissue of origin, are represented in Fig. 3 as box plots. With the exception of breast and lung, primary tumors always showed highest similarity with their cognate cell lines (t-test p-value<0.01). In particular, brain, leukemia, colon and ovary were the tissues with the most pronounced correspondence between tumors and cell lines. For breast and lung tumors the analysis indicates that not only their cognate cell lines, but also cell lines of different origins have comparable *Membranome* expression similarity.

### *Membranome*-driven pathways differentially regulated in primary tumors and cell lines

**Figure 4:**
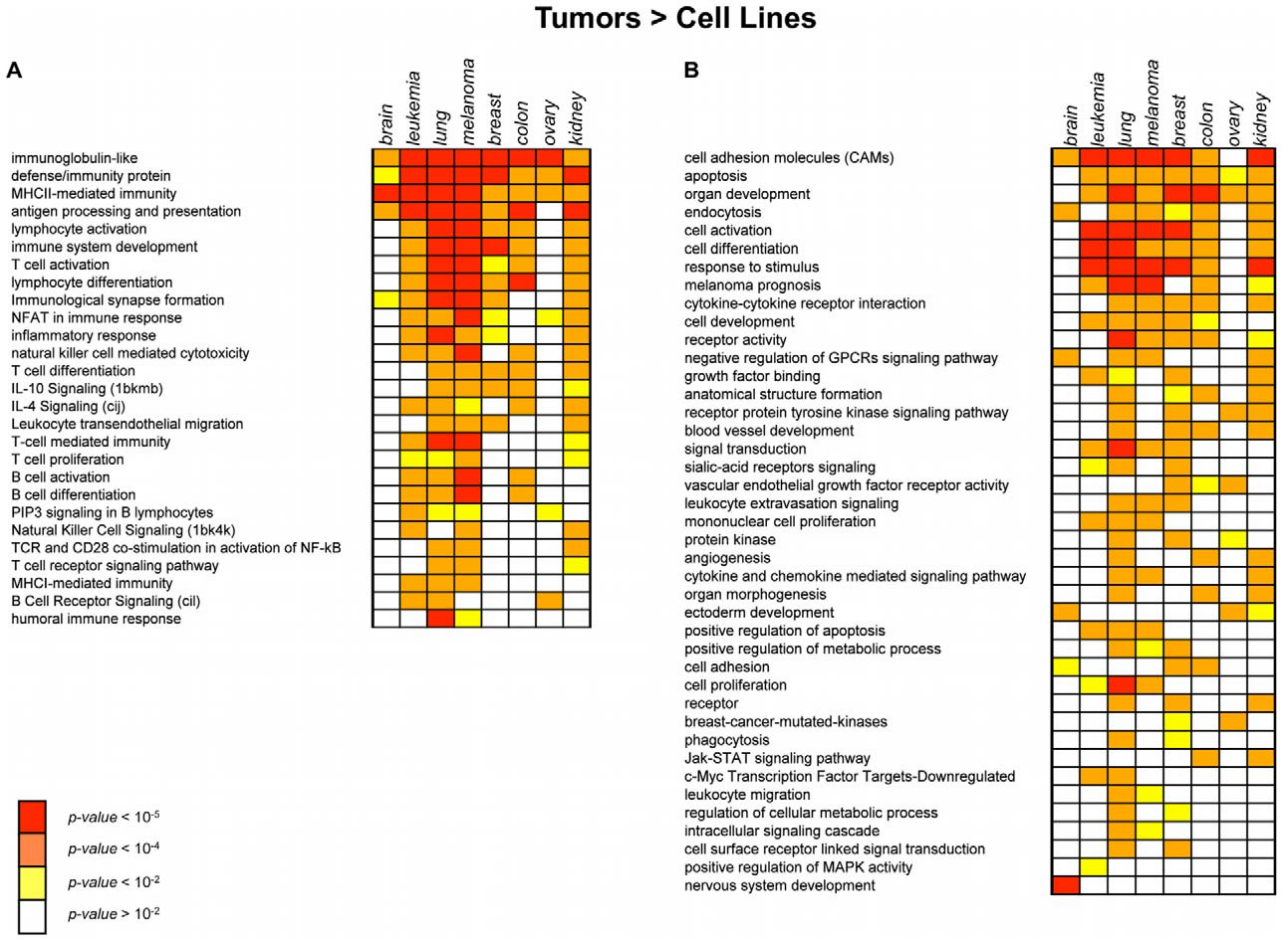
Gene sets enriched in genes up-regulated in tumors. Heatmap showing the gene sets significantly enriched in *Membranome* genes up-regulated in tumors as compared to cell lines of the same tissue origin. A) Immune-related pathways B) Other pathways. Gene set enrichment p-values, calculated using Fisher-exact test are represented by color codes (see legend).

**Figure 5:**
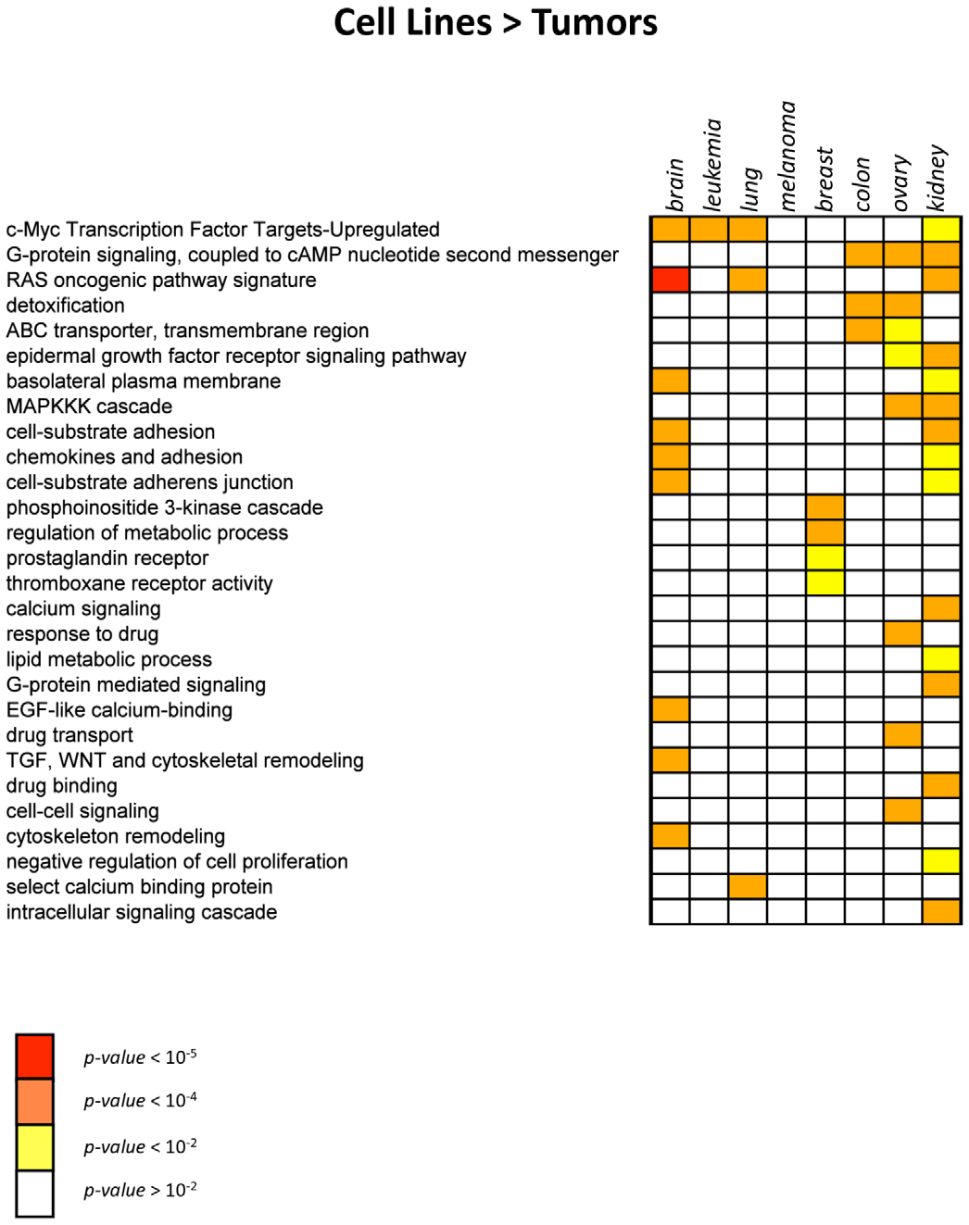
Gene sets enriched in genes down-regulated in tumors. Heatmap showing the gene sets significantly enriched in *Membranome* genes down-regulated in tumors as compared to cell lines of the same tissue origin. Gene set enrichment p-values, calculated using Fisher-exact test are represented by color codes (see legend).

To better characterize the differences between primary tumors and cell lines at the cell surface level, an analysis of the *Membranome* pathways differentially regulated in the two systems was performed. For each tumor type, differentially regulated genes in primary tumors and their cognate cell lines were identified by SAM (FDR<0.01). The resulting groups of up- and down-regulated genes were analyzed separately by using a gene set enrichment approach (see Methods). A representative extract of the results is illustrated in Fig. 4 and 5 (complete results are available in Table S4). Among the dominant themes up-regulated in primary tumors emerge those related to the immune response (Fig. 4A). These include “B-cell, T-cell and antibody mediated immunity”, “antigen presentation”, “NFAT in immune response”, “immunological synapse formation”, “regulation of T-cell proliferation”, “Natural killer cell mediated immunity”. Other themes generally up-regulated in primary tumors are those related to “cell adhesion”, “extracellular matrix”, “signal transduction”, “cell-cell communication”. Also the “cell differentiation” and “organ development” pathways appear also up-regulated in different tumor types (Fig. 4B).

As expected, more specialized pathways/gene sets are up-regulated in a more restricted manner. Examples are “nervous system development” and “melanoma prognosis”, specifically up-regulated respectively in brain and melanoma tumors.

Interestingly, the “breast cancer mutated kinases” gene set – composed of kinases genetically mutated in primary breast tumors [25] - appears to be up-regulated only in breast and ovary tumors, as compared to the corresponding cell lines. Overall, only a limited number of pathways and gene sets were found to be up-regulated in cell lines vs primary tumors and conservation of up-regulation was limited across cell lines of different origin (Fig. 5). Examples include the “c-myc transcription factor targets upregulated” (brain, leukemia, lung), the “RAS oncogenic pathway signature” (brain, lung, kidney) and the “G-protein signaling, coupled to cAMP” (“colon, ovary, kidney”). Of interest is also the up-regulation of pathways related to drug metabolism such as “detoxification”, “ABC transporter” (colon and ovary), “drug binding” (kidney) and “response to drugs” (ovary).

### *Membranome* tumor deregulated genes in primary tumors and cell lines

**Figure 6:**
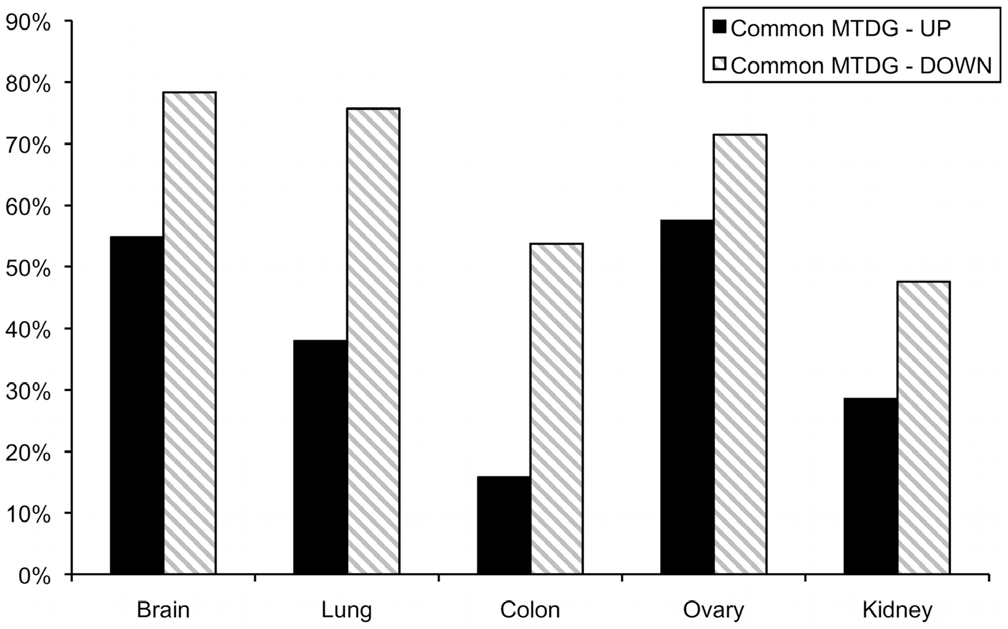
*Membranome* Tumor Deregulated Genes in tumors and cell lines. Percentages of the *Membranome* Tumor Deregulated Genes (MTDG) consistently deregulated in primary tumors and cell lines with the same origin. The number of MTDG in tumors and cell lines is reported in Table 2.

To further investigate on the nature of the similarities and differences between primary tumors and cell lines in the *Membranome* expression we considered also samples of normal origin in the study. We defined as MTDG (*Membranome* tumor deregulated genes), those *Membranome* genes up- or down-regulated in either primary tumor or cell line samples, as compared to normal samples with the same tissue origin. The analysis was restricted to those tissues for which cell lines, primary tumors and normal samples were available: brain, lung, colon, ovary and kidney. For each tissue, MTDG were identified in primary tumors and cell lines, using SAM (*FDR*<0.01) [26] (Table 2 and Table S3) and the percentages of MTDG with consistent regulation between primary tumors and the cell lines were computed (Table 2 and Fig. 6). The highest match was observed in brain, ovary and lung tissues, with 65%, 65% and 64%, respectively, of common MTDG between primary tumors and cell lines. Ovary, colon and kidney follow with 44% and 39%, respectively. When the percentages are instead analyzed separately for up- and down-regulated MTDG, higher values where consistently obtained for down-regulated MTDG. A significant portion of *Membranome* genes up-regulated in primary tumors therefore lose their de-regulation in cell lines, i.e. following immortalization and in the context of *in-vitro* growth conditions. Conversely, the majority of *Membranome* genes down-regulated upon tumor transformation maintain lower expression levels also in the cell lines. Noteworthy, tumors of different types always show the most significant overlap with the cell lines of same tissue origin (Table 3).

**Table 2:** Membranome differentially regulated genes (MTDG) in primary tumors and cell lines.

Data	Brain	Lung	Colon	Ovary	Kidney
Cell lines UP	353	183	48	334	179
Cell lines DOWN	528	503	311	363	418
Tumor tissue UP	363	158	76	193	273
Tumor tissue DOWN	277	366	227	207	355
Intersection: cell lines and tumor tissue UP	199(55%)	60(38%)	12(16%)	111(58%)	78(29%)
Intersection: cell lines and tumor tissue DOWN	217(78%)	277(76%)	122(54%)	148(71%)	169(48%)
Intersection: cell lines and tumor tissue UP or Down	416(65%)	337(64%)	134(44%)	259(65%)	247(39%)

The percentages of MTDG identified in primary tumors showing coherent regulation in the cell lines are shown in parentheses.

**Table 3:** −log10 Fisher exact test p-values of the overlaps between MTDG in primary tumors (T) and cell lines (CL).

	Brain (T)	Lung (T)	Colon (T)	Ovary (T)	Kidney (T)
**Brain (CL)**	**23.5**	0.0	0.0	0.0	0.0
**Lung (CL)**	0.0	**40.6**	2.8	0.0	0.0
**Colon (CL)**	0.0	8.8	**23.8**	0.0	0.0
**Ovary (CL)**	0.0	2.6	0.0	**30.3**	0.0
**Kidney (CL)**	0.0	0.2	0.1	0.0	**3.2**

The table shows that all the primary tumors analyzed have the highest statistically significant overlap with the cell lines originating from the same tumor type.

## Discussion

Characterization of general transcriptional similarities and differences between cell lines and primary tumors has been addressed by a variety of studies [27]–[32]. Higher proliferation rate and the adherent growth conditions of *in-vitro* cultured cell-lines appear to be the major factors clearly differentiating the two systems [33]. However, despite the crucial role of the cell surface in the cancer biology, and the common use of cell lines as an *in-vitro* model for cancer, little is known on how cell surface properties change when tumor cells move to *in-vitro* growth conditions. Here we examined the problem with a very focused perspective, specifically looking at genes codifying for plasma membrane proteins – the *Membranome*. These genes not only play a crucial role in the genesis and development of cancer, by mediating complex interactions between the tumor cells surface and the surrounding cellular environment [1], but constitute the target of election of monoclonal antibodies based therapies [2].

First we demonstrated that the expression of *Membranome* genes has greater power, as compared to the rest of the transcriptome, when used for the automatic classification of tumor samples according to their tissue of origin. This is also true for cell lines, although they are more difficult to classify and give rise to higher misclassification rates. These observations reinforce the role of *Membranome* genes determining the tumor specificity and indicate that much of the specificity of tumors originating from different tissues resides in their cell surface components. The higher promiscuity of cell lines in classification analysis mirrors - at a transcriptional level - the notion that *in-vitro* stabilized tumor cells have lost the tissue organization - and therefore the membrane characteristics - of the *in-vivo* tumor.

In order to quantify the degree to which cell lines are representative of their tumor of origin, with respect to *Membranome* expression, we have run a correlation analysis between primary tumors and cell lines. We showed that, with the exception of breast and lung, primary tumors show cell surface maximal similarity with the cell lines of same tissue origin (t-test p-value<0.01). In particular, brain, leukemia, colon and ovary were the tumors with the most pronounced correspondence, suggesting their membrane composition being mostly preserved in the cognate cell lines. The lack of maximal correlation between breast and lung cell lines with their respective tumors can probably be ascribed to their heterogeneous gene expression patterns, already pointed out by previous clustering analysis, in this case performed at the whole-genome level [8].

To understand which cell surface biological themes are differentially regulated between primary tumors and cell lines, a gene set enrichment analysis against a large sets of databases and cancer data extracted from the literature was performed.

This type of analysis is significantly more interpretable than a standard gene-level approach as it allows for a global overview of the cell surface processes differentiating the two systems, potentially hidden from a gene-centric perspective.

With gene set enrichment analysis lists of up- and down-regulated genes are translated into a more interpretable view of the biological pathways, which – as wholes - are differentially regulated in primary tumors and cell lines. Another important advantage lays in the fact that the perturbation of each pathway is quantified by an “aggregated” value, inferred from the statistical integration of dozens of genes taking part to the same pathway. This makes this analysis intrinsically more resistant to the presence of false positive/negative genes, which could potentially affect a “gene-centric” analysis, based on the evaluation of individual data points.

Among the dominant themes up-regulated in primary tumors emerge those related to the immune response, pathways known to be up-regulated in all tumors, regardless of their tissue of origin [27] (Fig. 4A). Tumor infiltrating lymphocytes (TIL) present in the extracted tumor samples are probably responsible for part of these molecular phenotypes. However, also pathways related to MHC class I antigen presentation emerge from the analysis, indicating an active role of tumor cells in the activation of immune response pathways and mirroring the complex interplay between tumor cells and TIL. We also observed the up-regulation of the “chemotaxis” and “cytokine and chemokine mediated signaling” pathways, respectively in five and three tumor types. Taken together these data are coherent with a recently proposed model of interaction between tumor and immune system cells [34]. The model suggests that TIL provide cytokines and growth factors necessary for tumor growth with tumor cells producing chemotactic factors that actively recruit mononuclear cells, mainly lymphocytes and macrophages, to tumor sites [34].

Other themes generally up-regulated in primary tumors are those related to “cell adhesion”, “extracellular matrix”, “signal transduction” and “cell-cell communication” (Fig. 4B). The up-regulation of many genes involved in these pathways apparently reflects the organization of primary tumor cells in tissues, in contrast to the altered environment of cells growing *in-vitro* in defined cell-culture media [35]–[37]. The “cell differentiation” and “organ development” pathways appear also up-regulated in different tumor types reflecting a general higher level of differentiation of primary tumor cells. Additional pathways/gene sets are instead up-regulated in a more tissue specific manner. Examples are “nervous system development” and “melanoma prognosis”, specifically up-regulated in brain and melanoma tumors, respectively. Interestingly, although brain tumors show up-regulation of some immune-related processes, many immune related gene sets do not show up. This divergence from other tumor types can possibly be explained by the particular characteristics of the CNS cellular environment, which influences its receptivity to immune activity. For example the existence of the blood-brain barrier (BBB), lower T-cell numbers within the CNS under normal circumstances and unconventional lymphatics [38].

The “breast cancer mutated kinases” gene set – composed of kinases found to be genetically mutated in primary breast tumors [25] - was found to be up-regulated only in breast and ovary tumors, as compared to cell lines. Both these tumors are originating from estrogen responsive tissues and are known to share hereditary genetic predisposition factors [39].

Only a limited number of pathways and gene sets were found to up-regulated in cell lines vs primary tumors. This is consistent with the results of the *Membranome* tumor deregulated genes (MTDG) analysis discussed below, showing that a significant portion of the *Membranome* loses its up-regulated state passing from *in-vivo* to *in-vitro* conditions. Noticeably, the gene sets we identified as up-regulated in cell lines, have limited conservation across cell lines of different origin (Fig. 5). These include the “c-myc transcription factor targets upregulated” (brain, leukemia, lung), the “RAS oncogenic pathway signature” (brain, lung, kidney) and the “G-protein signaling, coupled to cAMP” (“colon, ovary, kidney”). The up-regulation of these pathways is likely to reflect cell-line specific activation of signal transduction pathways through the cell surface and are related to the higher proliferation rate of the *in-vitro* cultures. Of interest is also the up-regulation of pathways related to drug metabolism such as “detoxification”, “ABC transporter” (colon and ovary), “drug binding” (kidney) and “response to drugs” (ovary). The differential regulation of these pathways can possibly underpin the different anticancer drug sensitivities observed *in-vitro* and *in-vivo* [27].

With the analysis of MTDG, we enquired whether *Membranome* genes deregulated in primary tumor samples as compared to their normal tissue counterparts retain their altered state also in the cell lines. This information is of key importance when using the cell lines as an *in-vitro* model for surface cancer targets. Examples are the screening of anticancer therapeutics targeting cell surface receptors [40] or the use of cell lines for the selection of cell-surface cancer specific mAbs from random peptide libraries [6], [41]. Importantly, a significant portion of MTDG over-expressed in primary tumors are lost in cell lines. Conversely, the majority of MTDG down-regulated upon tumor transformation are retained in *in-vitro* cultured cells (Fig. 6). The observation that cell lines tend to lose the tumor-specific gene up-regulations is in agreement with what previously reported at global transcriptional level [29]. Another interesting observation is that tumors of different origin always have the most significant overlap of MTDG with the cell lines originating from the same tissue (Table 3). This is true even for lung tumors, where the correlation analysis demonstrated a high level of similarity also with cell lines other than lung. It therefore appears that cell lines - despite some loss of the overall tumor characteristics - preferentially retain the tumor specific *Membranome* deregulation observed in primary tumors as compared to their normal counterparts.

As a further development of this study, the *Membranome* analysis at the protein level would be very useful to complement and validate our observations at the transcriptional level. In fact, mRNA abundances do not necessarily correspond to the levels of the protein functionally available and expressed on the cell surface. However, while recognizing the importance of this information for the detailed dissection of individual pathways, we believe the statistical approach that was undertaken in our study guarantees the general observations and conclusions to be valid also at the protein level. Indeed, despite single mRNA-protein levels divergences (high mRNA-low protein and vice-versa) can exist, their effects are expected to reciprocally compensate – and therefore to be strongly mitigated – in the context of a “global” scale analysis, one involving thousands of genes.

Additional comments need to be made regarding the samples we considered in the analysis. Our study has been constrained by the availability of transcriptional data sets publically available on a coherent microarray platform (the integration of data sets deriving from different technologies would have introduced too much noise in the meta-analysis). As a result, we created a meta-dataset, all based on the Affymetrix HG-U95Av2 platform, which to our knowledge was the platform covering the broadest spectrum of tumor samples. It encompasses 10 independent studies, covering a total of 409 human samples deriving from 8 different tissues. Additional tumors (e.g. sarcoma tumors, because of their particular biology involving the interactions with the extracellular matrix) and *in-vitro* tumor models (e.g. cell lines grown in three-dimensional conditions such as *mammospheres* or *neuroshperes*) could add further interest to our observations.

Using transcriptional data from a large set of primary tumors, normal tissue and cell lines of different origin we have demonstrated a central role of *Membranome* genes in characterizing the tumor phenotype. The comparative analysis of primary tumors and corresponding cell lines reemphasizes the caution that should applied when using these model systems in the study of the cancer. The presented results contribute to a more informed use of cell lines and interpretation of results with regards to specific aspects of tumor biology involving the cell surface.

## Materials and Methods

### Microarray data

Expression data for NCI60 cell lines were made publicly available through the Developmental Therapeutics Program of NCI/NIH. The NCI60 dataset includes data from 59 cell line. Cell culture growth conditions are described in [8]. The two cell lines of prostate origin (PC3 and DU-145 [8]) were not included because previous studies showed a low correlation with primary prostate tumors [30] as well as with other tumors [29]. We further removed the MDA-MB-435 cell line [8] because of its uncertain classification: originally considered as breast, it has also been reported to originate from melanoma [8], [29], [42].

No specific information is reported in the existing literature regarding the cell passage number at which cell lines were processed for microarray analysis. However, interesting information regarding this point can be found in the work of Ross and collaborators: *“[…]RNA samples from two cell lines (MCF7 breast and K562 leukaemia) were collected on three different occasions (at different passage numbers), then labelled, hybridized and scanned independently. These replicates (labeled MCF7 I, II and III, and K562 I, II and III) clustered side by side, with approximately the same degree of similarity as shown by the MDA-MB435/MDA-N pair […]”* [8].

These data, although limited to two cell lines only, point towards a relative transcriptional stability of these cell line samples across different passage numbers.

The set of primary tumors included 21 classic glioblastoma and anaplastic oligodendroglioma [15], 19 infiltrating ductal breast adenocarcinomas [19], 21 colorectal adenocarcinomas [19], 11 clear cell carcinoma of the kidney [19], 14 serous papillary ovarian adenocarcinomas [19], 72 leukemia samples (including 20 mixed-lineage leukemias, 24 acute lymphoblastic leukemias and 28 acute myelogenous leukemias) [11], 127 lung adenocarcinomas [12] and 9 melanoma tumors [16].

Normal tissue samples data were available for five different tissue types: brain [17], [18], colon [14], [19], kidney [13], [17]–[19], lung [12], [19] and ovary [18], [19]. All data are MIAME compliant and the raw data have been deposited in a MIAME compliant database. Expression data can be obtained from the following sources: [8], Developmental Therapeutics Program of NCI/NIH at http://dtp.nci.nih.gov/mtargets/download.html; [11], [12], [15], supplementary material available at http://www.broadinstitute.org/cgi-bin/cancer/datasets.cgi; [13], GEO accession GSE1563; [14], GEO accession GSE405; [16], supplementary material available at http://www.mskcc.org/genomic/ccsmsp/; [17], GEO accession GSE803; [18], GEO accession GSE96; [19], supplementary material available at http://public.gnf.org/cancer/epican/.

The meta-dataset deriving from the integration of the individual data sets described above represent to our knowledge the largest study publicly available based on the Affymetrix HG-U95Av2 array. More detailed information on samples included in this study is provided in Table S2.

### Data processing

All datasets were processed using the MAS5 algorithm implemented in R [43] and scaled to a trimmed mean value of 500. Expression values across technical replicates were averaged for lung tumors, brain normal, kidney normal and ovary normal samples. All arrays were normalized using a quantile normalization algorithm [44]. Finally, data was log 2 transformed prior to analysis.

### Classification of *Membranome* genes

A semi-automated procedure was applied to identify the human *Membranome*, here defined as the ensemble of all human genes coding for proteins integral to or covalently associated with the plasma membrane,

All human genes reported in the NCBI CCDS database (NCBI Build 37.1) [45]–[46] were surveyed using a combined analysis of the available Gene Ontology annotations [20] and the results of the Phobius algorithm for the prediction of trans-membrane domains and signal peptides [21]. The list of membrane protein genes thus created was manually revised to exclude proteins localized in intracellular compartments (false positives) and to include additional membrane-associated proteins known from literature (false negatives). These proteins were initially not included by the automated analysis because of missing annotation and/or lack of transmembrane domains, for example GPI–anchored proteins.

### Classification Analysis

To compute the ‘discriminative power’ of *Membranome* and Not-*Membranome* genes the PAM method (“Prediction Analysis of Microarrays”, PAM) [47] was applied to classify samples according to their tissue of origin.

PAM is based on nearest shrunken centroids classification and builds a classifier by identifying those genes that best characterize each group of samples. The size of the gene list used as the classifier, and the corresponding misclassification rate, depend on the shrinkage parameter Δ provided as input. PAM was run independently on primary tumors and cell lines using gene lists of decreasing sizes. Parallel analyses were performed using equally sized lists of *Membranome* and *Not-Membranome* genes, randomly chosen. For each list size (and therefore for each value of Δ) the analysis was run 1.000 times and the results of misclassification were averaged.

### Correlation analysis of cell lines and primary tumors

Cell lines and primary tumors were grouped according to their origin: brain, leukemia, lung, melanoma, breast, colon, ovary and kidney. All possible pairs of tumor and cell line samples were compared using the Pearson's correlation coefficient as the metric of similarity. Pearson's correlation values were computed between all tumor samples and all cell lines of two given groups (e.g. all lung tumors vs all breast cell lines). The resulting distributions of correlation values were represented as a box plots in Fig. 3. Mean values of correlation distributions were compared by Student's t-test with Bonferroni multiple comparison correction.

### Differential expression of Membranome genes

For each of the eight tissues in analysis, we computed the list of *Membranome* genes up- and down-regulated in the primary tumors as compared to the corresponding cell line, with the same tumor tissue origin (Table S3). For the five tissues for which also the normal samples were available (brain, colon, kidney, lung, ovary), we identified the MTDG (*Membranome* tumor deregulated genes) defined as *Membranome* genes up- or down-regulated in primary tumors or cell lines as compared to the normal samples of same tissue origin. Gene up- and down-regulations were in all cases assessed using the significance analysis of microarrays (SAM) [26], available as an R package. In a conservative approach we set FDR<0.01 for each pair wise comparison. For each tissue type, two lists of MTDG were compiled, respectively from the Tumor vs Normal and Cell line vs Normal comparisons.

The significance of the overlap between pairs of the lists was computed by using the Fisher's exact test and are reported in Table 3 as the negative log10 of the *p-value* obtained.

### Gene Set Enrichment Analysis

Lists of genes differentially up- or down- regulated were compared to annotated gene sets in order to identify functional classes that are significantly over-represented. Enrichment *p-values* were computed according to the Fisher's exact test. Gene sets were obtained from publicly publically available sources (Gene Ontology [20], KEGG [48], InterPro [49], Panther [22], Swissprot keywords, chromosome localization, miRNA targets identified after miRNA transfection [50], gene sets of relevance for cancer taken from several published sources [51]–[60]) and additional sources (GeneGo (GeneGo Inc., St Joseph, MI, USA), Ingenuity (Ingenuity Systems Inc, Mountain View, CA, USA), TRANSFAC [61]).

We decided to use and report the uncorrected *p-values* and not to correct for multiple testing. The latter decision was based on the observation of a very high degree of overlap between different gene sets. As a consequence, single tests performed on the individual gene sets are strongly dependent on each other, violating the assumption of independence required by standard correction methods such as ‘Bonferroni’, ‘Holm’ and ‘FDR’. Thus, in this context, standard correction for multiple testing would have resulted as too conservative. To be noted also that most of the pathways discussed have a *p-value* much lower than the standard threshold of 0.01.

## Supporting Information

Table S1 List of the 4,329 Human *Membranome* genes. Gene identifiers are based on the NCBI Build 37.1.
(0.56 MB XLS)

Table S2 Detailed description of the 409 samples analyzed in this study.
(0.10 MB XLS)

Table S3 Results of differential expression analysis (SAM, FDR<0.01). The file contains the complete list of 2,247 Affymetrix probes mapping to 1,701 *Membranome* genes represented on the HG-U95Av2 array. For each probe the table shows (from left to right): Affymetrix ID; Entrez GeneID; whether the probe is differentially expressed (SAM, FDR<0.01) in tumor-to-cell line, tumor-to-normal and cell line-to-normal comparisons; whether the probe is consistently differentially expressed in tumor-to-normal and tumor-to cell line comparisons. CL: cell line, T: primary tumor, N: normal sample.
(1.62 MB XLS)

Table S4 Results of gene set enrichment analysis. The file includes the 639 gene sets significantly enriched (p<0.05) in at least one of the comparisons tumor-to-cell line, tumor-to-normal and cell line-to-normal. For each gene set the table shows (from left to right): gene set ID; source of the gene set; gene set name; p-value enrichment (Fisher's exact test) for the genes differentially expressed (SAM, FDR<0.01) in each comparison; the number of overlapping genes; the Entrez Gene IDs of the overlapping genes. CL: cell line, T: primary tumor, N: normal sample.
(1.96 MB XLS)
